# Lactate as a Potential Biomarker of Sepsis in a Rat Cecal Ligation and Puncture Model

**DOI:** 10.1155/2018/8352727

**Published:** 2018-03-07

**Authors:** Xiaozhu Zhai, Zhengfei Yang, Guanghui Zheng, Tao Yu, Peng Wang, Xuefen Liu, Qin Ling, Longyuan Jiang, Wanchun Tang

**Affiliations:** ^1^Sun Yat-Sen Memorial Hospital, Sun Yat-Sen University, Guangzhou, China; ^2^Weil Institute of Emergency and Critical Care Research, School of Medicine, Virginia Commonwealth University, Richmond, VA, USA; ^3^Department of Emergency Medicine, Virginia Commonwealth University, Richmond, VA, USA

## Abstract

We attempted to investigate whether blood lactate is a useful biomarker for sepsis in a rat cecal ligation and puncture (CLP) model. Male Sprague-Dawley rats underwent approximately 75% cecum ligation and two punctures to induce high-grade sepsis. A lactate of 1.64 mmol/L (Youden score of 0.722) was selected as the best cutoff value to predict the onset of sepsis after CLP exposure; 46 of 50 rats who survived 24 hours after the CLP were divided into the L group (lactate < 1.64 mmol/L) and M group (lactate ≥ 1.64 mmol/L). In the M group, the animals had significantly higher murine sepsis scores and none survived 5 days post-CLP, and the rate of validated septic animals, serum procalcitonin, high mobility group box 1, blood urea nitrogen, alanine transaminase, cardiac troponin I, and the wet-to-dry weight ratio were significantly higher compared to the L group. Worsen PaO_2_/FiO_2_, microcirculations, and mean arterial pressure were observed in the M group. More severe damage in major organs was confirmed by histopathological scores in the M group compared with the L group. In conclusion, lactate ≥ 1.64 mmol/L might serve as a potential biomarker to identify the onset of sepsis in a rat CLP model.

## 1. Introduction

Sepsis has been newly defined as life-threatening organ dysfunction caused by a dysregulated host response to infection, which is a common cause of death of hospitalized patients with critical illness [1]. Despite numerous trials evaluating novel therapeutics in humans that were derived from animal sepsis models, few of them could be translated into patient improvements [2]. Among the many reasons, one could be the inappropriate application of animal models due to the loose inclusion criteria. To date, the outcomes of rodents who underwent insults to induce sepsis have been assessed mainly based on the mortality rate of the control (untreated) group [3], without consideration of the unique response of each animal to septic insult.

Rodent cecal ligation and puncture (CLP) model, which closely mimics the clinical course of intra-abdominal sepsis, has been widely used in septic research [4]. Although standardized surgical procedures are performed to facilitate a consistent outcome of CLP, a variable individual effect can be observed even in animals that initially receive identical insults [5]. Theoretically, if untreated, animals with no control of local infection would subsequently progress to sepsis, resulting in multiple organ failure, septic shock, and ultimately death. In fact, a certain proportion of animals subjected to CLP insults successfully walled off the infected area by local abscess formation, rarely developing sepsis and survive long-term [6, 7]. Thus, biomarkers should be investigated to distinguish septic animals from mild or nonresponders to CLP insults.

Limited studies have described biomarkers for assessing the acute onset of sepsis in rodents. The murine sepsis score (MSS) is a new robust system to predict the onset and endpoints of sepsis for each animal [8]. While based on simple observation, MSS is influenced by personal subjectivity and is currently not widely used. Serum procalcitonin (PCT) and interleukin-6 (IL-6) have been reported as diagnostic markers of sepsis [9], while they are unfortunately expensive or not readily available as ideal biomarkers. Microcirculation, the hallmark and motor of sepsis [10, 11], plays a pivotal role in the pathophysiology of sepsis and septic shock. Blood lactate (Lac) is a marker of abnormal microcirculation, reflective of tissue hypoperfusion and cellular hypoxia [12]. Additionally, the latest definitions for sepsis and septic shock (sepsis-3) emphasize the importance of Lac in reflecting the severity and early identification of sepsis in humans [1]. In practice, the determination of Lac is technically feasible, commonly used, and clinically available with a rapid turnaround time. Therefore, we hypothesize that Lac might be beneficial in a refined rodent model of sepsis induced by CLP. Here, we aim to explore the best cutoff value of Lac at 24 h post-CLP, the time point when the onset of sepsis often occurs in rodents [13, 14], and further validate the ability of Lac to distinguish sepsis from uncomplicated infection in a rat model of CLP.

## 2. Materials and Methods

### 2.1. Ethical Statement

All animals were cared for humanely and in compliance with the “Principles of Laboratory Animal Care” formulated by the National Society for Medical Research and the Guide for the Care and Use of Laboratory Animals prepared by the Institute of Laboratory Animal Resources and published by the National Institutes of Health (8th edition; Washington DC, National Academic Press, 2011). The protocol was approved by the Institutional Animal Care and Use Committee of the Tang Wanchun Laboratories of Emergency Critical Care Medicine, Sun Yat-Sen Memorial Hospital, Sun Yat-Sen University.

### 2.2. Animal Preparation

Healthy male Sprague-Dawley rats (weight, 460–520 g) were obtained from the Experimental Animal Center of Traditional Chinese Medicine University of Guangzhou. After inhaling a gradually increasing concentration of CO_2_ for brief anesthesia, all animals were anesthetized by an intraperitoneal injection of pentobarbital (45 mg/kg). The rectum temperature was continuously monitored using a temperature detector (BeneView T5, Mindary Bio-Medical Electronics Co. Ltd., Shenzhen, China). A PE-50 catheter was advanced into the abdominal aorta from the left femoral artery to measure the mean arterial pressure (MAP). This catheter was also used to provide samples for arterial blood gas, including the Lac and oxygenation index (PaO_2_/FiO_2_), and serum biochemical parameters such as procalcitonin (PCT), high mobility group box 1 (HMGB1), alanine transaminase (ALT), blood urea nitrogen (BUN), and cardiac troponin I (cTnI). Subsequently, CLP was performed as previously described [13]. In detail, after exposure, the cecum was gently extruded to ensure a uniform distribution of the internal contents in each rat. Approximately 75% of the cecum was ligated in a vertical position with 4–0 silk thread and double-punctured using an 18-gauge needle. The cecum was squeezed to assure expression of a small amount of fecal material. After wound closure, the animals were resuscitated via subcutaneous injection of warmed 0.9% normal saline (NS, 5 ml/kg) and extensively monitored until they regained independent mobility.

### 2.3. Experimental Protocol

This study was divided into two experimental stages. In the first stage of the study (7 days of follow-up), the best cutoff value of Lac to discriminate between septic and nonseptic animals was determined based on the receiver operating characteristic (ROC) curve. The second stage of the study (Lac-based sepsis stratification) was carried out to further validate the best cutoff value. A flowchart of the study is presented in Figure S1.

In the first stage, 50 rats were subjected to CLP to ensure a sufficient sample size at baseline to calculate a reliable standard deviation (SD) and at 24 hours post-CLP. Those who survived 24 hours post-CLP were stratified into a septic group (S group) and a nonseptic group (NS group) according to the thresholds for sepsis based on “The Third International Consensus Definitions for Sepsis and Septic Shock” (Supplement Table 1). Elements of sepsis, including infection, organ dysfunction, and hemodynamic perturbation, were investigated at 24 hours after animal preparation. The best cutoff value of Lac to discriminate between S group and NS group was obtained using a ROC curve and by quantifying the area under the curve (AUC).

In the second stage, a separate group of animals (*n* = 50) exposed to the same CLP was divided into two groups according to the level of Lac at 24 hours post-CLP. In the prospective stratification approach, the rats were sacrificed immediately or continuously monitored for 7 days to test the best cutoff value. Blood biochemistry, hemodynamics, sublingual microcirculation including perfused small vessel density (PVD) and microcirculatory flow index (MFI), and histopathological changes in major organs including the lung, kidney, liver, and heart were compared between subgroups. Additionally, the 7-day survival rate and MSS were continuously monitored at a 24-hour interval. The animals were euthanized for autopsy when signs of imminent death (i.e., inability to maintain an upright position/tremor and/or agonal breathing) were shown or at the end of the study by deep intraperitoneal anesthesia.

### 2.4. Thresholds for Sepsis

To date, the definition of sepsis is not well stated in veterinary medicine. Our septic thresholds for male Sprague-Dawley rats are based on the clinical diagnosis of sepsis [15], which mainly includes parameters of infection, organ dysfunction, and hemodynamic perturbation (Supplement Table 1). Briefly, elevated PCT and HMGB1 were required as markers of systemic inflammation caused by intraperitoneal infection. The infectious criteria plus at least two of the following parameters were sepsis. Serum BUN, ALT, and cTnI were detected as laboratory signs of renal, liver, and cardiac injury, respectively. Acute lung injury was suggested by PaO_2_/FiO_2_. MAP and Lac were monitored as reflections of hemodynamic perturbation.

### 2.5. Measurements

Unless stated, all measurements were performed at baseline and 24 hours post-CLP. Arterial blood pressure was recorded on a PC-based data acquisition system supported by WINDAQ software (DATAQ Instruments Inc., Akron, OH, USA). Arterial blood (0.1 ml) was drawn for blood gas analysis using a Radiometer ABL FLEX™ 80 (Radiometer, Copenhagen, Denmark).

### 2.6. Blood Bacterial Culture and Serum Biochemical Assays

Blood samples were drawn via the PE-50 catheter using sterile technique. In the first stage, 10 *μ*l of blood sample was on 5% sheep blood agar and incubated at 37°C for 24 hours under aerobic conditions to determine the bacterial load. Colony-forming units were quantified by manual counting. Approximately 0.6 ml of blood sample was collected for biochemical assays in two stages of the study. After centrifugation (1000 ×g, 15 minutes, 4°C), the serum was removed and stored at −80°C for further analysis. Serum PCT, HMGB1 (Cusabio Biotech Co. Ltd., Wuhan, China), and cTnI (Nanjing Jiancheng Bioengineering Institute, Nanjing, China) were detected using specific enzyme-linked immunoassay kits according to the manufacturer's instructions. Serum BUN and ALT were measured with a Hitachi 7180 biochemistry automatic analyzer (Hitachi, Co. Ltd., Tokyo, Japan).

### 2.7. Sublingual Microcirculation

In the second stage, sublingual microcirculation was visualized with the aid of a side stream dark-field imaging device (MicroScan; MicroVision Medical Inc., Amsterdam, Netherlands). MFI was quantified according to Boerma et al. [16]. The image was divided into four quadrants, and the predominant type of flow (absent = 0, intermittent = 1, sluggish = 2, and normal = 3) was assessed in small vessels (less than 20 *μ*m in diameter) in each quadrant. The MFI score represents the average value of the four quadrants. PVD was calculated as the number of small perfused vessels crossing the lines divided by the total length of the lines [17, 18]. The vessel size was measured with a micrometer scale superimposed on the video display. All recordings were analyzed by two independent observers.

### 2.8. Histological Examination Analysis

The lung, kidney, liver, and heart were harvested at 24 hours post-CLP in the second stage. Tissues were immediately fixed in 10% formaldehyde and then paraffin-embedded and sliced. The prepared tissue samples were stained with hematoxylin and eosin (HE) and observed under a light microscope for histological analysis. All slides were reviewed blindly and scored using a semiquantitative scoring system as previously described [19–21]. The histology injury scores were expressed as the sum of the individual scores.

### 2.9. Lung Wet-to-Dry Weight Ratio

To quantify the magnitude of pulmonary edema, lung tissues were harvested, the wet weight was immediately recorded, and the tissue was placed in an oven at 70°C for 48 hours until the weight was constant to record the dry weight. The wet-to-dry weight ratio was calculated as follows: W/D ratio = (wet weight − dry weight)/dry weight [22].

### 2.10. 7-Day Survival Rate and MSS

In the second stage, census of survival and MSS was continuously examined and confirmed by two investigators at a 24-hour interval for a total of 7 days. MSS was scored according to Shrum et al. (0 to 35 scale, normal = 0 and extremely severe = 35) [8].

### 2.11. Statistical Analysis

Statistical analyses were performed with SPSS 20.0 (SPSS Inc., Chicago, IL, USA). The 7-day survival curve was plotted using the Kaplan-Meier method and compared between groups with a log-rank test. The ROC curve was used to evaluate the prognostic accuracy of Lac and determine the sensitivity and specificity at selected cutoff values. The accuracy of the ROC-AUC test was rated as follows: 0.9–1, excellent; 0.8–0.9, good; 0.7–0.8, fair; 0.6–0.7, poor; and <0.6, not useful [23]. Normally distributed data were tested using the unpaired Student's *t*-test (with Welch correction for unequal variances whenever necessary). The Mann–Whitney *U* test was used when the data were not normally distributed. The rate of validated septic animals was expressed as a percentage and tested using the chi-square test. Group sizes reported for the evaluation of MSS varied over time, reflecting the mortality rate in animals that underwent CLP. Variables below the limit of detection were assigned a value that was equal to one-half of the lower limit of detection in the standard curve. The mean ± SD of differences would represent a 95% likelihood of an actual physiological change [15, 24]. *p* < 0.05 was considered statistically significant.

## 3. Results

### 3.1. Physiological Parameter Analysis and Defining Thresholds for Sepsis

The study was divided into two experimental stages. In the first stage of the study, baseline data obtained from 50 rats were retrospectively analyzed to calculate the SD (Supplement Table 2), of which 45 rats survived 24 hours post-CLP and were stratified into the S group (*n* = 27) and the NS group (*n* = 18) according to the thresholds for sepsis listed in Supplement Table 1.

### 3.2. Animal Stratification and Comparisons

No statistical differences in baseline measurements were observed between the S group and NS group. However, significantly higher serum PCT and HMGB1 were observed in the S group when compared with the NS group. Animals in the S group exhibited significantly worse organ functions than those in the NS group, as evidenced by the clearly higher levels of BUN, ALT, and cTnI and a lower ratio of PaO_2_/FiO_2_. Moreover, markedly lower MAP and higher levels of Lac were observed in the S group in comparison to the NS group. However, there was no significant difference in blood bacterial counts between the two groups (Table 1). Totally, 42.2% (19/45) of rats underwent CLP insults was found culture-positive (data not shown).

### 3.3. The Best Cutoff Value of Lac to Predict Sepsis

**Figure 1:**
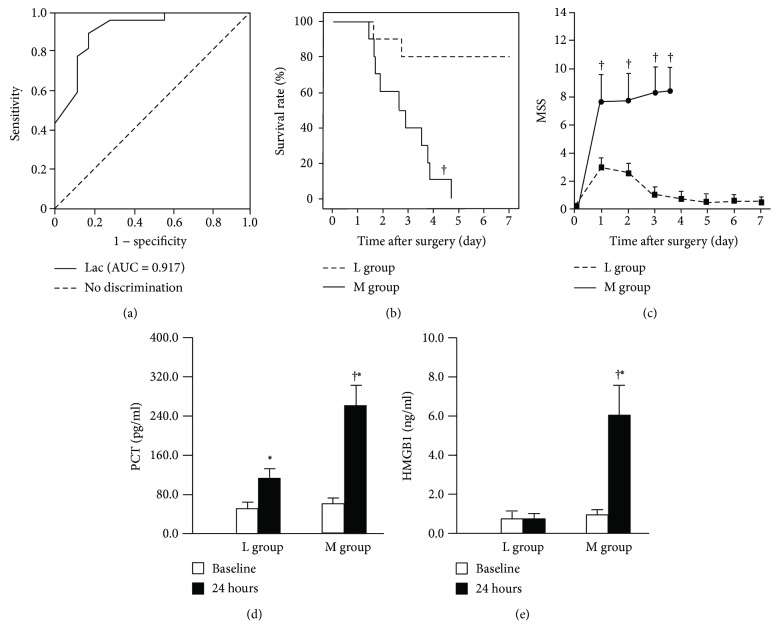
(a) Receiver operating characteristic (ROC) curve showed the best cutoff value of Lac to discriminate septic rats from nonseptic rats after CLP insult was 1.64 mmol/L (Youden score of 0.722). (b) The 7-day survival analysis (*n* = 10/each group). (c) The 7-day MSS. The group size reported for MSS data varied over time, reflecting the mortality rate (*n* = 10 at day 1, *n* ≥ 3 at other time points). ^†^ *p* < 0.01 versus L group. The serum levels of PCT (d) and HMGB1 (e) were significantly higher in animals in the M group compared with the L group, ^∗^ *p* < 0.01 versus baseline, ^†^ *p* < 0.01 versus L group.

The AUC (95% confidence interval) for Lac was 0.917 (83.3%–100.0%), which was excellent for in predicting the onset of acute sepsis (Figure 1(a)). A Lac of 1.64 mmol/L (Youden score of 0.722) was selected as the best cutoff value for rats that developed sepsis after CLP insult: the sensitivity and specificity of Lac were 88.9% and 83.3%, respectively.

### 3.4. Validation of the Best Cutoff Value

In the second stage of the study, animals that had survived for 24 hours (*n* = 46) were distributed into two groups according to the level of Lac: Lac < 1.64 group (L group, *n* = 18) and Lac ≥ 1.64 group (M group, *n* = 28). The two subgroups were randomly separated from the L and M groups, respectively: comparison of 7-day survival and MSS was observed in one subgroup (*n* = 10 in the L group; *n* = 10 in the M group); comparisons of blood biochemistry, hemodynamic abnormality, and histopathological changes were analyzed in the other subgroup (*n* = 8 in the L group; *n* = 18 in the M group).

**Figure 2:**
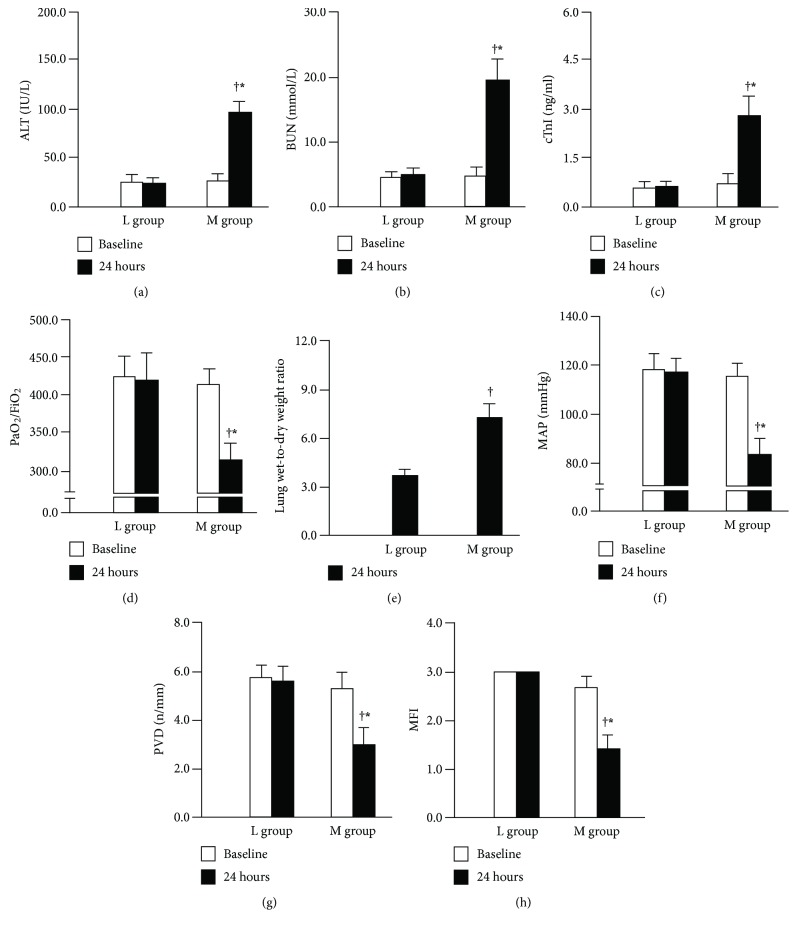
The serum ALT (a), BUN (b), and cTnI (c) and wet-to-dry weight ratio (e) of the M group were higher than the L group, while a lower PaO2/FiO2 (d) ratio was observed in the M group compared with the L group. ^∗^ *p* < 0.01 versus baseline, ^†^ *p* < 0.01 versus L group. MAP (f), PVD (g), and MFI (h) level were lower in the M group compared to the L group. ^∗^ *p* < 0.01 versus baseline, ^†^ *p* < 0.01 versus L group.

**Figure 3:**
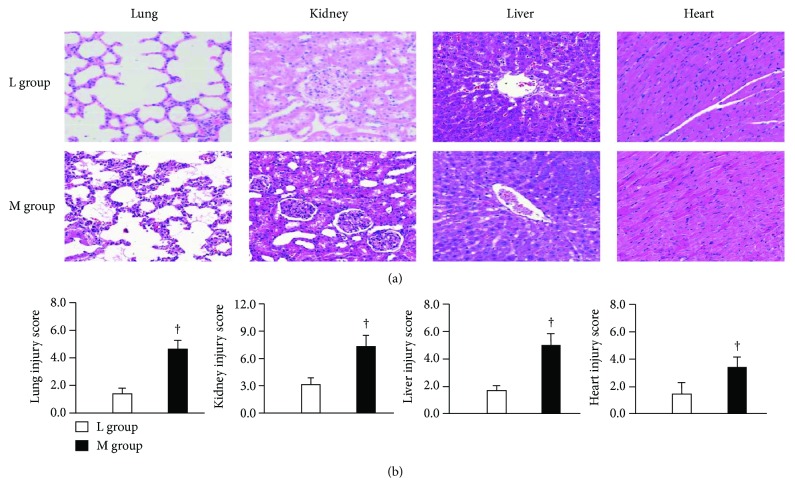
Histological examination (a) hematoxylin and eosin staining in major organs. Representative photographs are shown (original magnification ×20). (b) Histology injury scores of major organs. Pathology scores were evaluated as previously described in Materials and Methods. Lac: blood lactate; L group: group with Lac lower than 1.64 mmol/L at 24 hours after CLP (*n* = 3); M group: group with Lac equal to or more than 1.64 mmol/L at 24 hours after CLP (*n* = 3). ^†^ *p* < 0.01 versus L group.

None of the animals survived in the M group, whereas 8 out of 10 in the L group survived for 7 days after CLP (*χ* ^2^ = 11.99, *p* < 0.001) as shown in Figure 1(b). Moreover, a significantly lower MSS was observed in animals in the L group in comparison with the M group (Figure 1(c)). Progression to sepsis occurred in 88.9% (16/18) of the animals in the M group according to the thresholds for sepsis, while only 12.5% (1/8) developed sepsis in the L group (*p* < 0.01). The serum levels of PCT and HMGB1 were significantly higher in animals in the M group compared with the L group (Figures 1(d) and 1(e)). Higher serum BUN, ALT, cTnI, and wet-to-dry weight ratio and a lower PaO_2_/FiO_2_ ratio were observed in the M group compared with the L group (Figures 2(a)–2(e)). In comparison to the animals in the L group, MAP, PVD, and MFI were clearly poorer in the animals of the M group (Figures 2(f)–2(h)). In the M group, lung histopathology showed alveolar congestion and hyaline membrane formation, as well as neutrophil infiltration in the vessel walls; renal sections revealed bleb formation and tubular luminal debris; parenchymal cells demonstrated obvious vacuolization, and loss of organization and structure in liver tissues; and myocardial cellular edemas, small focal hemorrhages, and inflammatory cell infiltration were observed in the myocardia (Figure 3(a)). However, there were no or mild histological changes in the L group. Higher pathology scores which represented more severe damage in major organs were observed in the M group compared with the L group (all *p* < 0.01, Figure 3(b)).

## 4. Discussion

To date, no reliable biomarker has been utilized to individually evaluate the successful establishment of sepsis in a rodent CLP model, which might lead to, at least in part, the failure to translate the findings from animal models into meaningful changes in patient outcomes. Lac, a widely used and simple, easy-to-measure indicator, might be a potential biomarker for the improved identification of septic animals that are subjected to CLP. Therefore, we investigated the best cutoff value of Lac and further validated it in two separate experimental stages. In the first stage of the study, we found that Lac was excellent for prediction of the onset of acute sepsis (AUC = 0.917, 83.3%–100.0%), with an optimal cutoff value of 1.64 mmol/L (Youden score of 0.722). In the second stage of the study, we further confirmed that 1.64 mmol/L of Lac was a useful biomarker to discriminate sepsis from uncomplicated infection, which might be utilized to screen out septic animals after CLP insults.

We relied on the CLP model because it closely mimics the inflammatory, hemodynamic, and metabolic responses observed in human sepsis originating from the abdominal compartment. However, inherent variability persists in the CLP model owing to uncontrollable differences in animals and operators. Additionally, CLP cannot guarantee a constant leakage of fecal contents [25]. Theoretically, with 75% of the cecum ligated and punctured, rodents will progress rapidly to “high-grade” sepsis and die of overwhelming infection and multiple organ damage within 4 days without adjuvant therapies [14, 26] However, a series of studies have demonstrated that a considerable percentage (approximately 30–40%) of animals gradually recovered and survived at least 7 days after CLP [7, 27]. Consistently, our study demonstrated that 40.0% (8/20) of animals who underwent CLP as described above exhibited only a minor physiological disturbance, as reflected by a low MSS during experimental observation. Autopsy also confirmed the formation of their localized intra-abdominal abscess that was well tolerated by the rats.

In most successful experiments, the animals were included and administered the new therapeutics or agents before or shortly after CLP insult [28], only if the mortality in the control (untreated) group reached 60%–80%, a percentage that was usually adopted to mimic clinical mortality in severe sepsis [8, 29]. This evaluation method due to insufficient laboratory diagnostics overlooks the high variability in mortality that occurs in different laboratories and operators and the heterogeneity in the magnitude of the response to CLP in individual animals.

Indeed, numerous studies have been previously done in search of good predictive biomarkers of human sepsis mortality. While few are referred to be practically used in animal models of sepsis, especially in a rodent CLP model. HMGB1, a protein previously known only as a nuclear transcription factor, is now implicated as a mediator of delayed endotoxin lethality and systemic inflammation. PCT is a specific marker of bacterial infection in different patient groups and conditions, varying from neonatal sepsis to outpatients with respiratory complaints [30]. However, serum HMGB1 and PCT were generally investigated quantitatively using specific enzyme-linked immunoassay kits in the laboratory. It might take at least 24 hours to run ELISAs on 96-well plates, the whole process of which was relatively complicated, on the condition that all the serum samples were collected.

A hallmark of sepsis is an early onset of microcirculatory dysfunction. The development of microcirculation failure has been characterized as a concomitant increase in lactate levels and impaired microcirculatory perfusion [31]. An increased Lac level is a reflection of cellular dysfunction in sepsis, albeit recognizing that multiple factors, such as insufficient tissue oxygen delivery, impaired aerobic respiration, accelerated aerobic glycolysis, and reduced hepatic clearance, also contribute. Hyperlactatemia is, however, regarded as a reasonable marker of sepsis severity, with higher levels predictive of higher mortality [32]. Moreover, a recent study demonstrated that measurement of lactate is a key risk stratification tool to identify a distinct subgroup of severe sepsis patients that may require different treatment strategies [33]. Better still, Lac could be obtained simply and be available promptly within 5 minutes at a reasonable cost. Thus, the primary purpose during the first stage was to investigate the best cutoff value of Lac to discriminate animals who progressed to sepsis after CLP insult from those who did not. All elements of sepsis were included as thresholds to stratify animals that underwent CLP. At 24 hours post-CLP, we observed more severe infection and inflammation, worse hemodynamic abnormality, and more serious organ dysfunction in septic animals. Lac had a high AUC of 0.917 (95% CI: 83.3%–100.0%), supporting its excellent discriminatory power. A Lac level of 1.64 mmol/L (Youden score of 0.722) at 24 hours post-CLP was the best cutoff to predict septic animals. In the second stage of the study, 7-day mortality and MSS were significantly higher in the M group. In addition, the rate of validated septic animals was also clearly higher in the M group than in the L group. Similarly, more severe infection and host inflammatory response and worse organ function were observed in the M group. Furthermore, histopathological examination and macro/microcirculation parameters (MAP, PVD, and MFI) further confirmed that Lac equal to or greater than 1.64 mmol/L effectively excluded the possibility that the rat would be a mild or nonresponder to CLP insult.

There are several limitations in the present study. Firstly, we only used acute septic models in the present study, in which sepsis onset and progression to multiorgan failure occurred rapidly. Mild septic models characterized by the slow breakdown of organisms due to continuous smoldering sepsis [14] were not discussed in the present study. Secondly, only a 24-hour time point and its best cutoff value of Lac were investigated. According to previous reports, rats subjected to CLP as described above appear normal for approximately 10 hours after CLP and demonstrate hyperdynamic, hyperinsulinemic, and hypermetabolic states. They begin to show clinical signs of sepsis at around 12 hours following CLP. At approximately 16 hours, CLP-induced sepsis is marked by hypoglycemia, hypoinsulinemia, and hypodynamic circulation along with markedly increased lactate levels [13]. Moreover, the 24-hour time point is commonly selected to evaluate pathophysiological changes in models of acute sepsis [34–36]. Whether or not the best cutoff values of Lac at 12-hour or 48-hour time points still do benefit to the stratification for the CLP model need further study. New approaches such as wireless biotelemetry are expected to allow real-time detection of the acute physiological deterioration that occurs during sepsis [37]. Thirdly, sepsis is a dysregulated systemic host response to inflammatory triggers including infection and sterile causes such as burn and trauma. Higher bacterial growth was observed in animals at 24 hours post-CLP when compared with their baseline, while there was no significant difference in circulating bacteria between the septic group and nonseptic group. Besides, positive cultures can be detected in only 42.2% of rats that underwent CLP. Possible explanations include the lack of replication studies due to the limited blood samples, individual variability, insufficient sample size, and difference in sample size between groups. Therefore, the correlation between Lac levels and bacteria levels was not investigated in the present study. Finally, only the CLP model was utilized in the present study. Another clinically relevant rodent model to mimic human sepsis is colon ascendens stent peritonitis (CASP), in which a stent of certain diameter is surgically inserted into the ascending colon and causes continuous influx of enteric bacteria into the peritoneal cavity, while the acute systemic hyperinflammatory response in CLP-induced sepsis is more similar to the clinical sepsis. Moreover, the hemodynamic response to CASP-induced sepsis is not as well characterized. Moreover, CASP is a more difficult surgical procedure than CLP.

## 5. Conclusion

In summary, our data demonstrated that Lac is a good predictive biomarker in the present study, at least as good as PCT, IL-6, or HMGB-1. While the measurement of Lac is much faster and cheaper than these other biomarkers, Lac equal to or greater than 1.64 mmol/L could be used to predict the onset of acute sepsis and exclude the animal who would be a mild or nonresponder to the CLP insult. Utilization of Lac may not be limited to the CLP model used in this study because it may provide a uniform approach to assess disease severity and to compare and combine results from other animal models of sepsis as well as different research teams.

## Figures and Tables

**Table 1:** Comparison of parameters between the S group and NS group (mean ± SD).

Parameter	Time	NS group	S group
Body weight (g)	Baseline	484.6 ± 19.2	480.5 ± 15.0
	CLP 24 h	469.6 ± 16.1^∗^	464.8 ± 13.5^∗^

PCT (pg/ml)	Baseline	66.2 ± 11.1	62.2 ± 9.8
	CLP 24 h	82.8 ± 12.5^∗^	208.3 ± 44.5^∗^^#^

HMGB1 (ng/ml)	Baseline	0.6 ± 0.2	0.5 ± 0.2
	CLP 24 h	0.9 ± 0.3	5.5 ± 1.4^∗^^#^

BUN (mmol/L)	Baseline	4.9 ± 1.4	4.5 ± 1.4
	CLP 24 h	5.3 ± 0.9	15.8 ± 3.6^∗^^#^

ALT (UI/L)	Baseline	24.5 ± 7.7	27.6 ± 8.0
	CLP 24 h	28.3 ± 7.7	64.2 ± 11.1^∗^^#^

cTnI (ng/ml)	Baseline	0.4 ± 0.2	0.4 ± 0.1
	CLP 24 h	0.5 ± 0.2	2.1 ± 0.9^∗^^#^

PaO_2_/FiO_2_	Baseline	477.5 ± 33.0	473.9 ± 37.1
	CLP 24 h	471.9 ± 29.7	347.8 ± 40.5^∗^^#^

MAP (mmHg)	Baseline	119.8 ± 7.0	121.6 ± 7.9
	CLP 24 h	115.7 ± 12.5	84.4 ± 19.6^∗^^#^

Lac (mmol/L)	Baseline	0.7 ± 0.2	0.8 ± 0.3
	CLP 24 h	1.3 ± 0.4	2.2 ± 0.6^∗^^#^

Bacterial counts (Log cfu/ml)	Baseline	0	0
	CLP 24 h	3.78 ± 0.20^∗^	3.87 ± 0.23^∗^

CLP: cecal ligation and puncture; PCT: procalcitonin; HMGB1: high-mobility group box 1; BUN: blood urea nitrogen; ALT: alanine aminotransferase; cTnI: cardiac troponin i; PaO_2_/FiO_2_: oxygenation index; MAP: mean arterial pressure; Lac: blood lactate; cfu: colony-forming units. S group: animals that met the septic thresholds after CLP insult (*n* = 27); NS group: animals that did not meet the septic thresholds after CLP insult (*n* = 18). ^∗^ *p* < 0.01 versus baseline, ^#^ *p* < 0.01 versus NS group.

## Supplementary Materials

Supplementary 1 Figure S1: flow chart of the experimental protocol. CLP: cecal ligation and puncture; Lac: blood lactate. S group: animals met the septic threshold after CLP insult; NS group: animals did not meet the septic threshold after CLP insult; L group: group with Lac lower than the best cutoff value; M group: subgroups with Lac equal to or more than the best cutoff value.

Supplementary 2 Supplement Table 1: thresholds for sepsis. Supplement Table 2: baseline data in the first stage of the study.
