# Supplementary material for: Lactate as a Potential Biomarker of Sepsis in a Rat Cecal Ligation and Puncture Model
Source: Mediators Inflamm. 2018 Mar 7;2018:8352727. doi: 10.1155/2018/8352727 (PMC5863333; doi:10.1155/2018/8352727)
Supplement: Supplementary 2 — Supplement Table 1: thresholds for sepsis. Supplement Table 2: baseline data in the first stage of the study. [file 8352727.f2.docx]

**Supplement table 1: Thresholds for sepsis**

| **Infection** | |
| --- | --- |
| 1.Serum PCT >84.5 pg/mL | 1 or more |
| 2.Serum HMGB1 >0.9 ng/mL |  |
| **Organ dysfunction and hemodynamic perturbation** | |
| 1. PaO_2_/FiO_2_ <403.7 | 2 or more |
| 2. BUN >7.5 mmol/L |  |
| 3. ALT >42.5 UI/L |  |
| 4.cTnI >0.7 ng/mL |  |
| 5. MAP <105.7 mmHg |  |
| 6. Lac >1.3 mmol/L |  |
| **Infection plus 2 parameters of organ dysfunction and hemodynamic perturbation indicate sepsis** | |

PCT, procalcitonin; HMGB1, high mobility group box 1; BUN, blood urea nitrogen; ALT, alanine aminotransferase; cTnI, cardiac troponin i; PaO_2_/FiO_2_, oxygenation index; MAP, mean arterial pressure; Lac, blood lactate.

**Supplement table 2: Baseline data in the first stage of the study (Mean ± SD)**

| Parameter | Baseline (n = 50) |
| --- | --- |
| PCT, pg/ml | 63.6 ± 10.4 |
| HMGB1, ng/ml | 0.6 ± 0.2 |
| ALT, UI/L | 26.5 ± 8.0 |
| BUN, mmol/L | 4.6 ± 1.5 |
| cTnI, ng/mL | 0.4 ± 0.1 |
| PaO_2_/FiO_2_ | 475.2 ± 35.7 |
| MAP, mmHg | 121.0 ± 7.7 |
| Lac, mmol/L | 0.8 ± 0.3 |

PCT, procalcitonin; HMGB1, high mobility group box 1; BUN, blood urea nitrogen; ALT, alanine aminotransferase; cTnI, cardiac troponin i; PaO_2_/FiO_2_, oxygenation index; MAP, mean arterial pressure; Lac, blood lactate.
